# Deep learning-derived biological age from preoperative chest radiographs predicts mortality following surgical and transcatheter procedures for structural heart disease beyond EuroSCORE II

**DOI:** 10.1093/ehjdh/ztag112

**Published:** 2026-07-20

**Authors:** Era Stambollxhiu, Miriam Kumpf, Maximilian-Niklas Bonk, Lisa C Adams, Marcus Makowski, Martin Hadamitzky, Markus Krane, Keno K Bressem, Oliver Deutsch

**Affiliations:** Institute for Diagnostic and Interventional Radiology, TUM School of Medicine and Health, Klinikum Rechts der Isar, TUM University Hospital, Ismaninger Str. 22, Munich 81675, Germany; Institute for Diagnostic and Interventional Radiology, TUM School of Medicine and Health, Klinikum Rechts der Isar, TUM University Hospital, Ismaninger Str. 22, Munich 81675, Germany; Department of Cardiovascular Surgery, TUM School of Medicine and Health, German Heart Centre Munich, Munich, Germany; Institute for Diagnostic and Interventional Radiology, TUM School of Medicine and Health, Klinikum Rechts der Isar, TUM University Hospital, Ismaninger Str. 22, Munich 81675, Germany; Institute for Diagnostic and Interventional Radiology, TUM School of Medicine and Health, Klinikum Rechts der Isar, TUM University Hospital, Ismaninger Str. 22, Munich 81675, Germany; Institute for Cardiovascular Radiology and Nuclear Medicine, TUM School of Medicine and Health, German Heart Center Munich, Munich, Germany; Department of Cardiovascular Surgery, TUM School of Medicine and Health, German Heart Centre Munich, Munich, Germany; Institute for Diagnostic and Interventional Radiology, TUM School of Medicine and Health, Klinikum Rechts der Isar, TUM University Hospital, Ismaninger Str. 22, Munich 81675, Germany; Institute for Cardiovascular Radiology and Nuclear Medicine, TUM School of Medicine and Health, German Heart Center Munich, Munich, Germany; Department of Cardiovascular Surgery, TUM School of Medicine and Health, German Heart Centre Munich, Munich, Germany

**Keywords:** Biological age, Chest radiography, Structural heart disease, Risk stratification, EuroSCORE II, STS-PROM, Transcatheter aortic valve implantation, Artificial intelligence

## Abstract

**Aims:**

Accurate risk stratification before structural heart disease interventions is essential for clinical decision-making. Traditional risk models, such as the European System for Cardiac Operative Risk Evaluation II (EuroSCORE II) and Society of Thoracic Surgeons Predicted Risk of Mortality (STS-PROM), were designed for surgical patients and show inconsistent performance in transcatheter cohorts. Biological age, reflecting cumulative physiological decline, may offer prognostic value beyond chronological age and established risk scores.

**Methods and results:**

In this retrospective study of 1269 patients [non-transcatheter aortic valve implantation (TAVI) *n* = 751, TAVI *n* = 518] treated at the German Heart Center Munich, biological age was estimated from pre-operative chest radiographs using CXR-Age, a validated deep learning model. Analyses were conducted separately for surgical (non-TAVI) and transcatheter (TAVI) groups. For 30-day mortality, biological age outperformed EuroSCORE II in both subgroups [area under the receiver operating characteristic curve (AUC): non-TAVI 0.874 vs. 0.785, *P* < 0.001; TAVI 0.952 vs. 0.745, *P* = 0.004] and remained independently predictive after adjustment [TAVI OR 1.58 per year, 95% confidence interval (CI) 1.27–2.12]. While STS-PROM was the strongest single predictor for non-TAVI patients (AUC 0.949), it was similar to EuroSCORE II for TAVI patients (AUC 0.729). Notably, patients whose biological age exceeded their chronological age by more than 10 years faced higher major complication rates (17.3% vs. 9.2%; *P* = 0.016).

**Conclusion:**

Biological age distinguished risk across both populations, suggesting that deep learning-based biological age estimation from routine chest radiographs could serve as an automated, accessible complement to existing risk models.

## Graphical Abstract

**Graphical Abstract:**
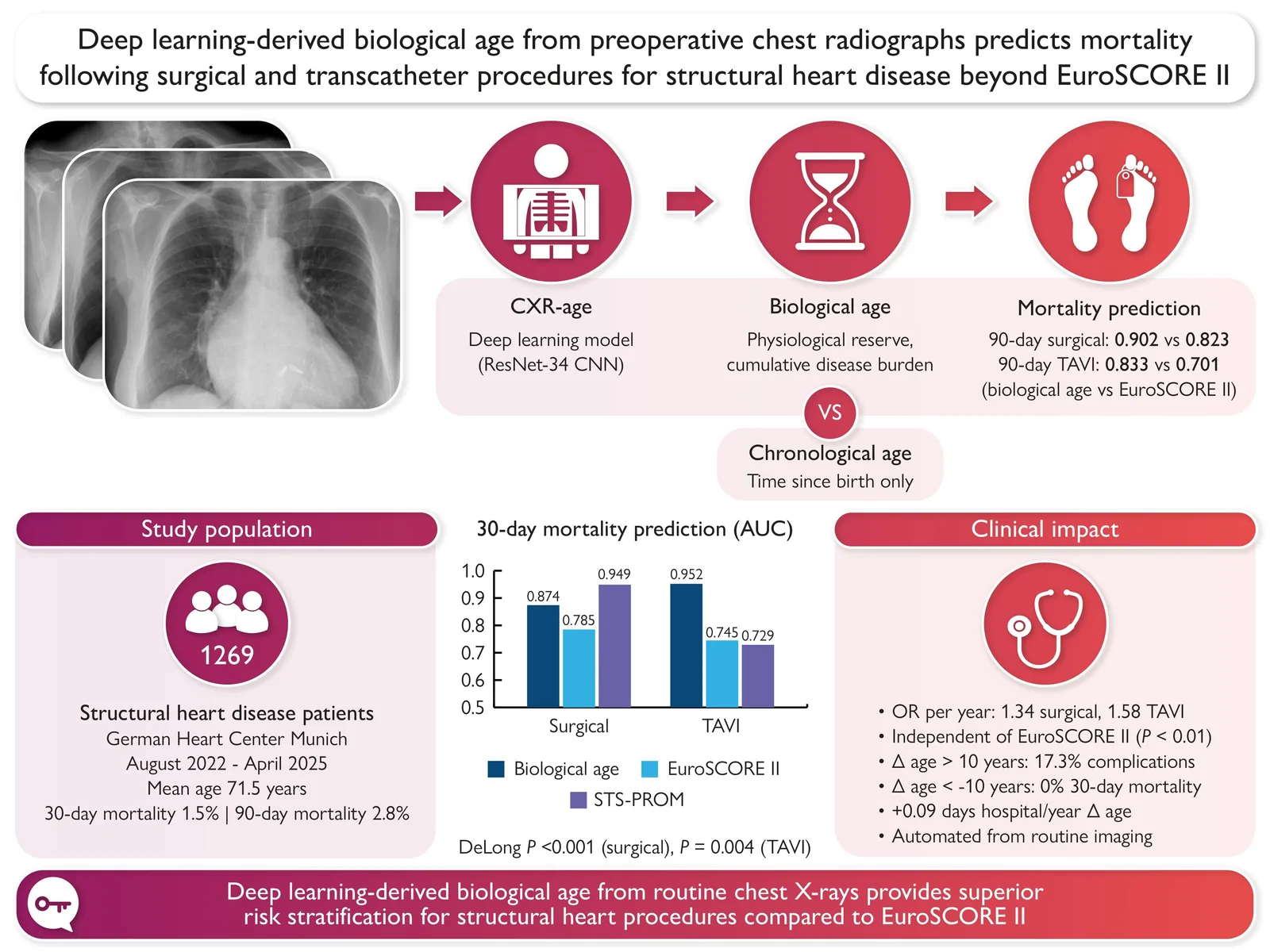

## Introduction

Cardiac surgery and transcatheter cardiac interventions carry a substantial perioperative risk, and accurate risk prediction is central to informed consent, resource allocation, and clinical decision-making. The European System for Cardiac Operative Risk Evaluation II (EuroSCORE II), introduced in 2012, remains the most widely adopted pre-operative risk model in European cardiac surgical practice,^1^ while the Society of Thoracic Surgeons Predicted Risk of Mortality (STS-PROM) score is the established standard in North America.^2–4^ However, the predictive performance of both scores has been variable across populations and time periods, with several studies documenting significant miscalibration in both European and non-European cohorts.^5–9^

A key limitation of conventional risk scores is their reliance on chronological age and discrete clinical variables, which cannot capture the heterogeneity in physiological reserve among patients of similar age. Two 75-year-old patients undergoing valve replacement may differ profoundly in their capacity to tolerate procedural stress. This variation is better described by biological age, which reflects the cumulative burden of disease and individual ageing trajectories rather than time since birth.^10–12^ The frailty syndrome, widely recognized as a predictor of adverse cardiac surgical outcomes, represents a clinical manifestation of accelerated biological ageing, yet formal frailty assessments are time-consuming and require specialized expertise, limiting routine adoption.^13–16^

Recent advances in deep learning have enabled automated estimation of biological age from routine medical imaging.^17^ CXR-Age, a convolutional neural network developed by Raghu *et al*., estimates biological age from chest radiographs by integrating radiographic features, including cardiac silhouette morphology, vascular calcification, pulmonary parenchymal changes, and musculoskeletal alterations.^18^ Trained on over 100 000 chest radiographs and validated against mortality data from large screening trials, CXR-Age has demonstrated superior prediction of all-cause and cardiovascular mortality compared with chronological age across diverse populations.^18–20^ Chest radiography is universally obtained during pre-operative evaluation, making CXR-Age an attractive candidate for integration into existing clinical workflows without additional testing or cost.

The purpose of this study was to evaluate whether deep learning-derived biological age from pre-operative chest radiographs predicts short-term mortality following surgical and transcatheter procedures for structural heart disease and to compare its predictive performance against the two most widely used clinical risk scores, EuroSCORE II and STS-PROM, within procedurally defined subgroups.

## Methods

### Study population

This retrospective cohort study screened 1881 patients aged 18 years or older who underwent surgical or transcatheter procedures for structural heart disease at the German Heart Center Munich between August 2022 and April 2025. Eligible patients underwent procedures for structural heart disease, including aortic, mitral, or tricuspid valve disease requiring surgical or transcatheter intervention. Patients undergoing isolated coronary artery bypass grafting (CABG) without a concomitant structural heart procedure were excluded. Isolated CABG was excluded because the CXR-Age model, although predictive of all-cause mortality in screening populations, was developed and validated for general cardiopulmonary ageing rather than coronary artery disease specifically,^18^ and because the perioperative risk profile of isolated CABG differs sufficiently from that of structural heart procedures to warrant evaluation in a separate, dedicated study.^2–4^ Patients undergoing combined CABG and structural heart procedures were retained and are reported within the Combined Procedures category (see Supplementary material online, *Table S1*).

Pre-operative posteroanterior chest radiographs were obtained from the institutional picture archiving and communication system. Patients without a pre-operative posteroanterior chest radiograph obtained at the institute were excluded. After quality control, 1269 patients with a unique pre-operative radiograph were included.

Each patient is represented only once in the analytical dataset. The case-level identifier in the institutional registry uniquely indexes each surgical or transcatheter encounter, and no patient was re-entered for a separate later procedure during the study window. Concomitant procedures performed during a single operation are captured under combined procedure categories [e.g. Combined Procedures and transcatheter aortic valve implantation (TAVI) combined with transcatheter mitral implantation], so that each patient contributes only a single record. Prior sternotomy was documented from the pre-operative chest radiograph and is reported in the baseline characteristics (*Table 1*).

**Table 1:** Baseline characteristics of the study cohort

Characteristic	Value
No. of patients	1269
Chronological age (years)	71.5 ± 12.7
Biological age (years)	70.3 ± 7.5
Age delta (biological − chronological)	−1.1 ± 8.3
EuroSCORE II (%)	5.2 ± 6.4
STS-PROM (%)	2.2 ± 4.3
30-day mortality	19 (1.5%)
90-day mortality	35 (2.8%)
Pre-operative pacemaker/ICD	81 (6.4%)
Prior sternotomy	15 (1.2%)
Foreign material visible	99 (7.8%)
Indwelling catheter	25 (2.0%)

Values are mean ± SD or *n* (%). Radiographic findings (pacemaker/ICD, prior sternotomy, foreign material, indwelling catheter) were documented from the pre-operative chest radiograph.

ICD, implantable cardioverter-defibrillator; STS-PROM, Society of Thoracic Surgeons Predicted Risk of Mortality.

Clinical outcome data, including 30-day mortality, 90-day mortality, length of hospital stay, length of intensive care unit stay, pacemaker implantation, neurological events, severe bleeding, and reoperation for bleeding, were extracted from electronic medical records. Pre-operative risk was assessed using the EuroSCORE II^1^ and the STS-PROM score.^2–4^

### Image acquisition and processing

Chest radiographs were retrieved using the Digital Imaging and Communications in Medicine (DICOM) protocols. DICOM files were converted to Portable Network Graphics format with standardized pre-processing. A board-certified radiologist with 8 years of experience (K.K.B.) performed manual quality control on all radiographs using a custom web-based annotation tool, excluding lateral projections and retaining only the frontal posteroanterior views suitable for analysis. For patients with multiple radiographs from the same examination, only the last pre-operative image was retained. After quality control, 1269 frontal chest radiographs were included.

### Biological age estimation

Biological age was estimated using CXR-Age, a validated convolutional neural network previously described by Raghu *et al*.^18^ The model employs a ResNet-34 architecture and was developed in two stages: initial training on over 100 000 chest radiographs from publicly available datasets (CheXpert, PadChest, and NIH CXR-14) to predict chronological age,^21–24^ followed by fine-tuning on data from the Prostate, Lung, Colorectal and Ovarian Cancer Screening Trial to estimate biological age as a predictor of all-cause mortality.^25^ Input images were resized to 224 × 224 pixels and normalized using ImageNet statistics. Test-time augmentation was applied to improve prediction robustness. The model outputs a continuous biological age estimate in years.

### Statistical analysis

All mortality analyses were performed within two pre-specified subgroups defined by procedural approach and were not pooled: a TAVI subgroup (transcatheter aortic valve implantation, including TAVI performed with sternotomy or cardiopulmonary bypass and TAVI combined with transcatheter mitral implantation) and a non-TAVI subgroup comprising all open surgical procedures, together with transcatheter mitral and tricuspid interventions. The TAVI vs. non-TAVI dichotomy was chosen because TAVI is the dominant transcatheter category and the procedure for which EuroSCORE II is most widely documented to underperform.^26^ Within each subgroup, EuroSCORE II, STS-PROM, and CXR-Age were compared in the complete-case cohort, where all three scores were available. A pooled, full-cohort comparison was not performed because the surgical and transcatheter populations differ too fundamentally to be combined.

Descriptive statistics were calculated for biological age, chronological age, and the age delta (Δ, biological minus chronological age). Correlation between biological and chronological age was assessed using Pearson’s correlation coefficient, and agreement using the Bland–Altman analysis. Within each subgroup, the discrimination of biological age for 30-day and 90-day mortality was compared against EuroSCORE II, STS-PROM, and chronological age using the area under the receiver operating characteristic curve (AUC). Pairwise AUC comparisons used the DeLong test for correlated curves.^27^ The incremental value of biological age beyond each clinical score, and vice versa, was assessed using likelihood-ratio tests. Internal validation used bootstrap resampling with 1000 iterations to estimate optimism-corrected AUCs. Calibration was assessed using Brier scores, scaled Brier scores, and calibration intercept and slope.

Univariable and multivariable logistic regression models for 30-day and 90-day mortality were constructed within each subgroup, including single-score models and combined models pairing biological age with EuroSCORE II or STS-PROM. Associations between age delta and resource utilization (hospital and intensive care unit length of stay) were examined using linear regression within each subgroup. Post-operative complications were analysed within each subgroup using logistic regression for biological age adjusted for EuroSCORE II. The Cochran–Armitage test assessed linear trends across ordered age-delta categories. A two-sided *P* value of < 0.05 was considered statistically significant. Analyses were performed using R version 4.3 with the tidyverse, pROC, and broom packages.

### Ethics

This study was approved by the institutional review board of the Technical University of Munich (reference number: 2026-99-W-CT). Due to the retrospective design using routinely collected clinical data, individual informed consent was waived.

## Results

### Patient characteristics

A total of 1269 patients were included. The mean chronological age was 71.5 years (SD ± 12.7), and the mean biological age was 70.3 years (SD ± 7.5); the mean age delta was −1.1 years (SD ± 8.3), indicating that on average the cohort appeared slightly younger than its chronological age (see Supplementary material online, *Figure S1*). Overall, 30-day mortality was 1.5%, and 90-day mortality was 2.8% (*Table 1*).

The non-TAVI subgroup (*n* = 751) and the TAVI subgroup (*n* = 518) differed substantially in their baseline profiles. TAVI patients were older (mean 80.2 vs. 65.4 years) and had higher EuroSCORE II (6.8% vs. 4.1%) and STS-PROM (2.9% vs. 1.4%) values. On average, TAVI patients appeared biologically younger than their chronological age (mean age delta −5.9 years), whereas non-TAVI patients appeared biologically older than their chronological age (+2.2 years). Thirty-day mortality was similar between subgroups (TAVI 1.4%, non-TAVI 1.7%), as was 90-day mortality (3.6% vs. 3.2%). Non-TAVI patients had longer hospital stays (median 10 vs. 5 days) and intensive care unit stays (median 1 vs. 0 days) and higher rates of reoperation (5.3% vs. 2.3%), neurological events (2.1% vs. 0.8%), and severe bleeding (5.7% vs. 0.0%), whereas pacemaker implantation was more frequent after TAVI (8.5% vs. 4.0%) (*Table 2*).

**Table 2:** Baseline characteristics and outcomes by procedural subgroup

Characteristic	Non-TAVI (*n* = 751)	TAVI (*n* = 518)
Chronological age (years)	65.4 ± 12.0	80.2 ± 7.6
Biological age (years)	67.6 ± 7.7	74.2 ± 5.3
Age delta (years)	2.2 ± 8.0	−5.9 ± 6.2
EuroSCORE II (%)	4.1 ± 5.5	6.8 ± 7.1
STS-PROM (%)	1.4 ± 4.1	2.9 ± 4.4
30-day mortality	12 (1.7%)	7 (1.4%)
90-day mortality	20 (3.2%)	15 (3.6%)
Hospital stay (days), median [IQR]	10 [8–14]	5 [4–7]
ICU stay (days), median [IQR]	1 [1–2]	0 [0–1]
New pacemaker	30 (4.0%)	44 (8.5%)
Reoperation	40 (5.3%)	12 (2.3%)
Neurological event	15 (2.1%)	4 (0.8%)
Severe bleeding	40 (5.7%)	0 (0.0%)

Values are mean ± SD, median [IQR], or *n* (%). Severe bleeding denotes Grades 2–3 (serious or life-threatening). Neurological event denotes stroke, transient ischaemic attack, or other cerebrovascular event.

ICU, intensive care unit; IQR, interquartile range; STS-PROM, Society of Thoracic Surgeons Predicted Risk of Mortality; TAVI, transcatheter aortic valve implantation.

Transcatheter aortic valve implantation was the most frequent procedure (*n* = 515), followed by isolated aortic valve replacement (*n* = 242) and mitral with or without tricuspid valve surgery (*n* = 248). Procedure-specific characteristics are detailed in Supplementary material online, *Table S1*.

### Biological and chronological age

**Figure 1:**
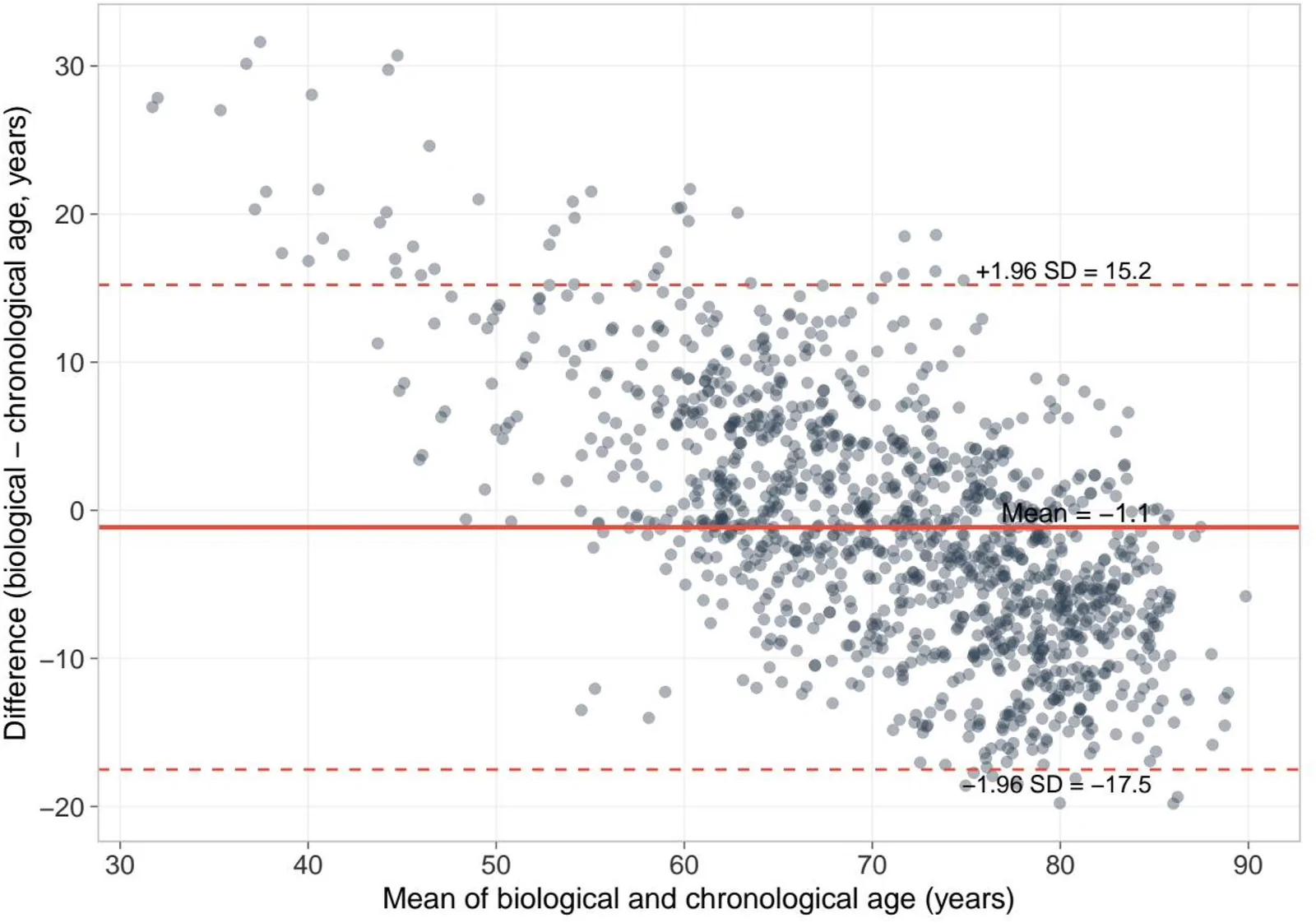
Bland–Altman plot showing agreement between biological and chronological age across the full cohort. Horizontal lines indicate the mean difference (−1.1 years) and the 95% limits of agreement (−17.5 to +15.2 years).

Biological age correlated strongly with chronological age (r = 0.77, *P* < 0.001) (see Supplementary material online, *Figure S2*). The Bland–Altman analysis demonstrated a mean difference of −1.1 years with 95% limits of agreement from −17.5 to +15.2 years, indicating substantial individual variation in the biological–chronological age discrepancy (*Figure 1*).

### Mortality prediction

**Figure 2:**
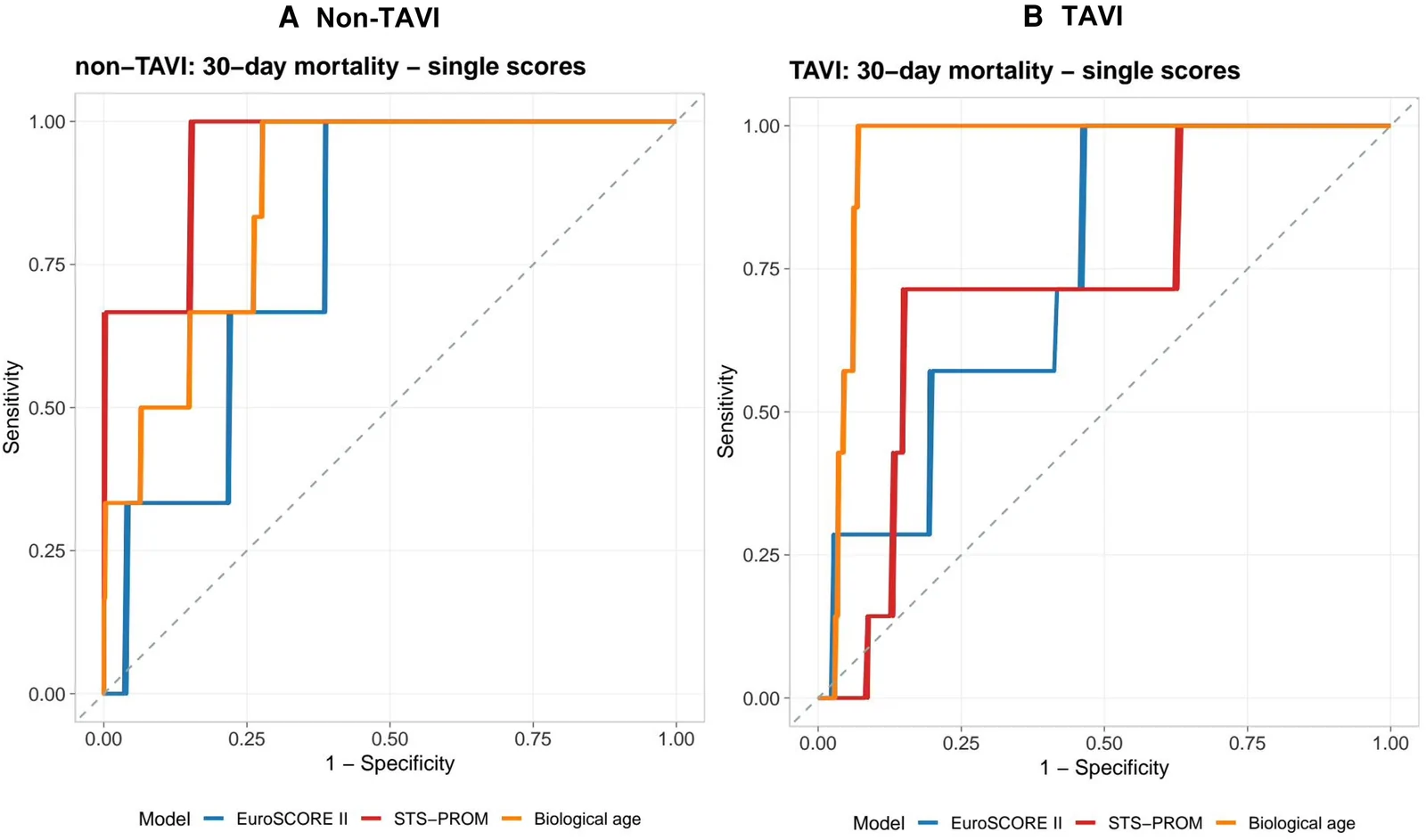
Receiver operating characteristic curves for 30-day mortality by procedural subgroup. (*A*) Non-TAVI subgroup and (*B*) TAVI subgroup, comparing EuroSCORE II, STS-PROM, and biological age (CXR-Age). Biological age was the strongest single predictor in the TAVI subgroup, whereas STS-PROM was strongest in the non-TAVI subgroup. EuroSCORE II, European System for Cardiac Operative Risk Evaluation II; STS-PROM, Society of Thoracic Surgeons Predicted Risk of Mortality; TAVI, transcatheter aortic valve implantation.

Within each procedural subgroup, biological age outperformed EuroSCORE II for 30-day mortality. In the non-TAVI subgroup, biological age achieved an AUC of 0.874 [95% confidence interval (CI) 0.773–0.975] vs. 0.785 (95% CI 0.658–0.912) for EuroSCORE II (DeLong *P* < 0.001); discrimination was comparable to chronological age (0.786; biological vs. chronological *P* = 0.115). STS-PROM was the strongest single predictor in this subgroup (AUC 0.949, 95% CI 0.886–1.000), exceeding both EuroSCORE II (*P* < 0.001) and biological age (*P* = 0.006). In the TAVI subgroup, biological age was by far the strongest single predictor (AUC 0.952, 95% CI 0.930–0.974), far exceeding EuroSCORE II (0.745, 95% CI 0.600–0.891; *P* = 0.004) and chronological age (0.880; *P* = 0.071), whereas STS-PROM (0.729, 95% CI 0.546–0.912) performed no better than EuroSCORE II (*P* = 0.09) and was significantly inferior to biological age (*P* = 0.018) (*Figure 2*; *Table 3*).

**Table 3:** Discrimination (AUC) for mortality by procedural subgroup

Model	Non-TAVI AUC (95% CI)	TAVI AUC (95% CI)
**30-day mortality**		
EuroSCORE II	0.785 (0.658–0.912)	0.745 (0.600–0.891)
STS-PROM	0.949 (0.886–1.000)	0.729 (0.546–0.912)
Biological age	0.874 (0.773–0.975)	0.952 (0.930–0.974)
Chronological age	0.786 (0.614–0.958)	0.880 (0.806–0.954)
EuroSCORE II + biological age	0.876 (0.777–0.975)	0.953 (0.931–0.975)
STS-PROM + biological age	0.926 (0.834–1.000)	0.952 (0.930–0.975)
**90-day mortality**		
EuroSCORE II	0.823 (0.719–0.927)	0.701 (0.609–0.792)
STS-PROM	0.954 (0.908–1.000)	0.709 (0.597–0.820)
Biological age	0.902 (0.821–0.982)	0.833 (0.710–0.956)
Chronological age	0.830 (0.693–0.968)	0.755 (0.649–0.860)
EuroSCORE II + biological age	0.901 (0.821–0.981)	0.834 (0.710–0.958)
STS-PROM + biological age	0.936 (0.859–1.000)	0.833 (0.710–0.956)

Subheading rows (30-day, 90-day mortality) span the predictor models below them. Pairwise DeLong comparisons (30-day): non-TAVI, EuroSCORE II vs. biological age *P* < 0.001, STS-PROM vs. biological age *P* = 0.006, STS-PROM vs. EuroSCORE II *P* < 0.001, biological vs. chronological age *P* = 0.115; TAVI, EuroSCORE II vs. biological age *P* = 0.004, STS-PROM vs. biological age *P* = 0.018, STS-PROM vs. EuroSCORE II *P* = 0.90, biological vs. chronological age *P* = 0.071.

AUC, area under the receiver operating characteristic curve; CI, confidence interval; EuroSCORE II (European System for Cardiac Operative Risk Evaluation II); STS-PROM, Society of Thoracic Surgeons Predicted Risk of Mortality; TAVI, transcatheter aortic valve implantation.

The 90-day results were concordant. Biological age outperformed EuroSCORE II in both subgroups (non-TAVI 0.902 vs. 0.823, *P* < 0.001; TAVI 0.833 vs. 0.701), while STS-PROM again achieved the highest discrimination among single scores in the non-TAVI subgroup (0.954) but not in the TAVI subgroup (0.709) (see Supplementary material online, *Figure S3*; *Table 3*).

In univariable logistic regression, each 1-year increase in biological age was associated with a higher 30-day mortality in both subgroups [non-TAVI odds ratio (OR) 1.33, 95% CI 1.14–1.60, *P* < 0.001; TAVI OR 1.59, 95% CI 1.28–2.13, *P* < 0.001], whereas EuroSCORE II was not a significant predictor in either subgroup (non-TAVI *P* = 0.129; TAVI *P* = 0.090). Biological age remained independently predictive after adjustment for EuroSCORE II (non-TAVI OR 1.34, 95% CI 1.13–1.65, *P* = 0.002; TAVI OR 1.58, 95% CI 1.27–2.12, *P* < 0.001), with EuroSCORE II being non-significant in the combined models. When biological age was combined with STS-PROM, STS-PROM remained the dominant predictor in non-TAVI patients (OR 1.20, *P* < 0.001; biological age *P* = 0.329), whereas biological age remained dominant in TAVI patients (OR 1.62, *P* < 0.001; STS-PROM *P* = 0.573). Results for 90-day mortality were concordant (*Table 4*).

**Table 4:** Logistic regression for 30-day and 90-day mortality by subgroup

Model/predictor	Non-TAVI OR (95% CI), *P*	TAVI OR (95% CI), *P*
**30-day mortality**		
EuroSCORE II (per %)	1.06 (0.95–1.13), 0.129	1.05 (0.98–1.10), 0.090
STS-PROM (per %)	1.24 (1.15–1.38), <0.001	1.04 (0.84–1.15), 0.621
Biological age (per year)	1.33 (1.14–1.60), <0.001	1.59 (1.28–2.13), <0.001
Chronological age (per year)	1.13 (1.03–1.27), 0.020	1.33 (1.12–1.65), 0.003
EuroSCORE II + bio: EuroSCORE II	0.98 (0.78–1.09), 0.833	1.02 (0.91–1.10), 0.677
EuroSCORE II + bio: biological age	1.34 (1.13–1.65), 0.002	1.58 (1.27–2.12), <0.001
STS-PROM + bio: STS-PROM	1.20 (1.10–1.35), <0.001	0.95 (0.72–1.11), 0.573
STS-PROM + bio: biological age	1.11 (0.91–1.40), 0.329	1.62 (1.29–2.21), <0.001
**90-day mortality**		
EuroSCORE II (per %)	1.07 (0.98–1.13), 0.043	1.03 (0.98–1.08), 0.171
STS-PROM (per %)	1.23 (1.14–1.35), <0.001	1.02 (0.88–1.11), 0.733
Biological age (per year)	1.37 (1.19–1.64), <0.001	1.32 (1.17–1.53), <0.001
Chronological age (per year)	1.17 (1.06–1.30), 0.002	1.18 (1.07–1.33), 0.004
EuroSCORE II + bio: EuroSCORE II	1.00 (0.84–1.08), 0.953	0.99 (0.91–1.05), 0.863
EuroSCORE II + bio: biological age	1.38 (1.18–1.66), <0.001	1.33 (1.17–1.54), <0.001
STS-PROM + bio: STS-PROM	1.16 (1.08–1.29), <0.001	0.95 (0.78–1.08), 0.541
STS-PROM + bio: biological age	1.22 (1.02–1.51), 0.040	1.34 (1.18–1.55), <0.001

Subheading rows span the models below them. Single-score rows are univariable; rows labelled ‘EuroSCORE II + bio’ and ‘STS-PROM + bio’ report the two predictors from the corresponding bivariable model.

OR, odds ratio; CI, confidence interval; bio, biological age.

Likelihood-ratio testing confirmed this complementary pattern. Biological age added significant information beyond EuroSCORE II in both subgroups (non-TAVI χ^2^ = 13.48, *P* < 0.001; TAVI χ^2^ = 21.06, *P* < 0.001). In the non-TAVI subgroup, biological age added little beyond STS-PROM (χ^2^ = 0.88, *P* = 0.348), while STS-PROM added strongly beyond biological age (χ^2^ = 22.00, *P* < 0.001); in the TAVI subgroup the reverse held, with biological age adding strongly beyond STS-PROM (χ^2^ = 23.32, *P* < 0.001) and STS-PROM adding nothing beyond biological age (χ^2^ = 0.50, *P* = 0.481) (see Supplementary material online, *Table S5*). Bootstrap validation confirmed the stability of these estimates, with minimal optimism (see Supplementary material online, *Table S4*), and calibration metrics were adequate across models (see Supplementary material online, *Table S3*). Combined models pairing biological age with EuroSCORE II or STS-PROM did not materially exceed the better single score within each subgroup (see Supplementary material online, *Figure S4*).

### Post-operative complications and outcomes by age delta

Within each subgroup, biological age modelled as a continuous variable and adjusted for EuroSCORE II was associated with 30-day mortality (non-TAVI OR 1.19 per year, 95% CI 1.05–1.35, *P* = 0.007; TAVI OR 1.61, 95% CI 1.24–2.27, *P* = 0.001) and with major complications in the TAVI subgroup (OR 1.14, 95% CI 1.02–1.27, *P* = 0.019). In the non-TAVI subgroup, the association with major complications was in the same direction but did not reach significance (OR 1.05, 95% CI 1.00–1.09, *P* = 0.058). Biological age was not significantly associated with neurological events, severe bleeding, pacemaker implantation, or reoperation in either subgroup; several of these endpoints had too few events for stable estimation (*Table 5*).

**Table 5:** Biological age and post-operative outcomes by subgroup

Outcome (events)	Non-TAVI OR (95% CI), *P*	TAVI OR (95% CI), *P*
30-day mortality	1.19 (1.05–1.35), 0.007 (*n* = 12)	1.61 (1.24–2.27), 0.001 (*n* = 7)
Major complication	1.05 (1.00–1.09), 0.058 (*n* = 88)	1.14 (1.02–1.27), 0.019 (*n* = 21)
Neurological event	1.10 (0.99–1.23), 0.081 (*n* = 15)	Not estimated (*n* = 4)
Severe bleeding	1.05 (0.98–1.12), 0.163 (*n* = 40)	Not estimated (*n* = 0)
New pacemaker	0.96 (0.89–1.04), 0.356 (*n* = 27)	1.02 (0.95–1.10), 0.539 (*n* = 44)
Reoperation	0.98 (0.92–1.05), 0.547 (*n* = 39)	1.10 (0.96–1.26), 0.175 (*n* = 12)

Odds ratios are per 1-year increase in biological age, modelled as a continuous variable and adjusted for EuroSCORE II within each subgroup.

Outcomes with no or very few events (TAVI neurological events, TAVI severe bleeding) could not be estimated reliably.

OR, odds ratio; CI, confidence interval.

Stratification by age-delta category across the full cohort revealed a clinically meaningful gradient in major complications. Patients whose biological age exceeded their chronological age by more than 10 years (*n* = 122) had a major complication rate of 17.3%, compared with 9.2% in age-matched patients (−5 to +5 years, *n* = 556) and 6.9% in biologically younger patients (< −10 years, *n* = 173) (Cochran–Armitage trend *P* = 0.016). Trends in 30-day and 90-day mortality across age-delta categories were not statistically significant (*P* = 0.453 and *P* = 0.706, respectively), although patients appearing biologically much younger had no 30-day deaths (*Table 6*; Supplementary material online, *Figures S5*–*S6*).

**Table 6:** Clinical outcomes by age-delta category (full cohort)

Age-delta category	*n*	30-day mortality	90-day mortality	Major complication
Much younger (< −10)	173	0.0%	1.5%	6.9%
Younger (−10 to −5)	260	3.2%	5.4%	8.0%
Age-matched (−5 to +5)	556	0.9%	3.3%	9.2%
Older (+5 to +10)	158	2.7%	3.0%	8.8%
Much older (> +10)	122	1.9%	2.2%	17.3%

Cochran–Armitage trend tests: 30-day mortality *P* = 0.453; 90-day mortality *P* = 0.706; major complication *P* = 0.016.

Age delta, biological age minus chronological age, in years.

### Resource utilization

**Figure 3:**
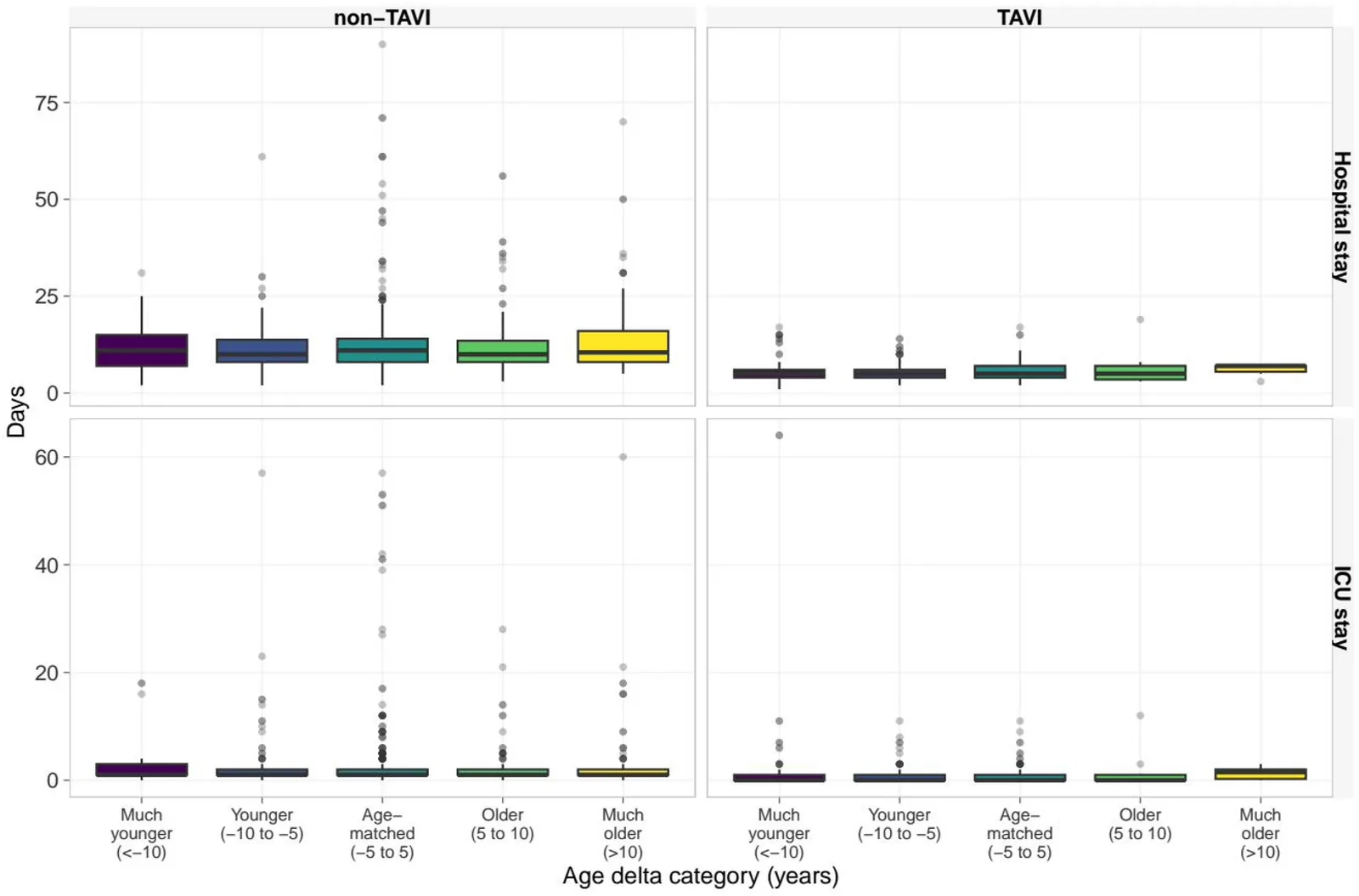
Hospital and ICU length of stay by age-delta category, shown separately for the non-TAVI and TAVI subgroups. The association between higher age delta and longer hospital stay was confined to the non-TAVI subgroup. ICU, intensive care unit; TAVI, transcatheter aortic valve implantation.

Associations between age delta and length of stay were modest and confined to the non-TAVI subgroup. Each 1-year increase in age delta was associated with 0.09 additional days of hospital stay in non-TAVI patients (95% CI 0.01–0.18, *P* = 0.032) but not in TAVI patients (β 0.01, 95% CI −0.02–0.05, *P* = 0.549). No significant association with intensive care unit length of stay was observed in either subgroup (*Figure 3*; Supplementary material online, *Table S2*).

## Discussion

This study demonstrates that deep learning-derived biological age from pre-operative chest radiographs outperforms EuroSCORE II for predicting short-term mortality after both surgical and transcatheter procedures for structural heart disease. Because EuroSCORE II was developed in surgical populations and was not designed for transcatheter procedures,^1,26^ a pooled comparison would partly reflect this mismatch; we therefore report all analyses within stratified TAVI and non-TAVI subgroups rather than in a combined cohort. CXR-Age outperformed EuroSCORE II in both subgroups, including the non-TAVI subgroup in which EuroSCORE II is the established standard.^1^ This finding indicates that the predictive value of biological age estimated from chest radiography is not driven solely by the inclusion of transcatheter cases and reflects a genuine signal of physiological reserve that is captured by routine pre-operative imaging.

Within each subgroup, biological age was numerically superior to chronological age for mortality discrimination, but this difference did not reach statistical significance (e.g. 30-day mortality in the TAVI subgroup: AUC 0.952 vs. 0.880; DeLong *P* = 0.071). Both measures clearly exceeded EuroSCORE II. This pattern suggests that much of the prognostic information carried by CXR-Age in this cohort is shared with chronological age, while the radiographic features quantified by the model add incremental value that is most apparent in the regression models, where biological age carried a larger per-year OR than chronological age and remained independently predictive after adjustment for the clinical scores. These observations reinforce the limitations of treating age as a simple linear predictor in surgical risk assessment, without implying that imaging-derived age fully supplants chronological age.

The addition of STS-PROM as a second comparator clarified the relative strengths of each score. STS-PROM, the established North American standard, was the strongest predictor in the non-TAVI subgroup, in which it is specifically calibrated, and in this group, CXR-Age added little discriminatory information beyond it. In the TAVI subgroup, the pattern reversed: STS-PROM performed no better than EuroSCORE II, whereas CXR-Age was by far the strongest predictor, and STS-PROM added nothing beyond it. The limited diagnostic accuracy of EuroSCORE II in transcatheter populations has previously been demonstrated in large TAVI cohorts,^26^ and the Society of Thoracic Surgeons score shows the same limitation: in a contemporary series of 3270 patients undergoing transcatheter aortic valve replacement, STS-PROM discriminated poorly for 30-day mortality (AUC 0.64), and even a dedicated transcatheter score (the STS/ACC Transcatheter Valve Therapy score) reached only 0.68, with no surgical risk stratum exceeding 0.70.^28^ Our subgroup analyses confirm this pattern: both EuroSCORE II and STS-PROM showed only modest discrimination in the transcatheter subgroup, whereas biological age estimated from chest radiographs retained strong predictive performance in both subgroups, suggesting that CXR-Age captures procedure-agnostic physiological reserve rather than features specific to surgical risk. Taken together, CXR-Age and STS-PROM are complementary: STS-PROM is the strongest single predictor in surgical patients, whereas CXR-Age is the only score that performs well in both surgical and transcatheter populations.

Unlike STS-PROM, a proprietary score that requires ∼30 manually entered clinical and laboratory variables and whose underlying algorithm is not openly available,^2–4^ CXR-Age is derived automatically from a single routine pre-operative chest radiograph. In our cohort, it matched STS-PROM in surgical patients and outperformed it in transcatheter patients while requiring no manual data entry, suggesting that biological age from chest radiography is a readily scalable complement to existing clinical risk scores.

These results extend a growing body of evidence supporting CXR-Age as a mortality predictor. In the original validation study, Raghu *et al*. demonstrated that CXR-Age predicted all-cause and cardiovascular mortality better than chronological age in screening populations.^18^ Lee *et al*. confirmed these findings in over 36 000 Asian individuals,^19^ and Chandra *et al*. showed that CXR-Age correlates with coronary calcium scores, cardiovascular risk factors, and frailty measures.^20^ The present study is the first to apply CXR-Age to perioperative risk prediction across surgical and transcatheter procedures for structural heart disease, a setting in which the consequences of inaccurate risk estimation are immediate and high stakes.

Stratification by age delta revealed an outcome gradient with direct clinical relevance: patients whose biological age exceeded their chronological age by more than 10 years had the highest major complication rate, nearly double that of age-matched patients, while those appearing biologically younger had no 30-day deaths. These patterns mirror the frailty literature. In the FRAILTY-AVR study, Afilalo *et al*. demonstrated that frailty was a significant predictor of death and disability following aortic valve replacement, with the Essential Frailty Toolset providing incremental prognostic value beyond established risk scores.^14^ CXR-Age offers a practical advantage over formal frailty assessment, as biological age can be estimated within seconds from routinely obtained imaging, without additional time, training, or specialized assessments. The association between age delta and hospital length of stay in the non-TAVI subgroup, although modest (0.09 additional days per year), is consistent with this interpretation.

From a clinical implementation perspective, chest radiography is universally performed during pre-operative evaluation. The CXR-Age algorithm can be applied to standard DICOM images with minimal pre-processing, generating a biological age estimate within seconds. This information could be incorporated into routine pre-operative discussions, supporting shared decision-making between patients and surgical teams. For patients identified as having accelerated biological ageing, targeted prehabilitation strategies may improve perioperative resilience.^29,30^

### Limitations

This study has several limitations. The single-centre retrospective design limits generalizability and warrants external validation. Event rates were low (30-day mortality 1.5%, 90-day mortality 2.8%), and the small number of deaths within the complete-case subgroups widened the CIs for some estimates, particularly the STS-PROM AUCs in the surgical subgroup. STS-PROM could not be computed for procedures without an applicable STS model and was applied off-label in TAVI patients. Although CXR-Age performed well in both subgroups for mortality prediction, the spectrum of post-operative complications differs systematically between surgical and transcatheter approaches; vascular complications dominate after transcatheter procedures, whereas bleeding, sternal wound complications, and prolonged ventilation are more characteristic of open surgery. Subgroup-specific evaluation of CXR-Age against complication-specific endpoints will require larger, dedicated cohorts. Formal frailty assessments were not available for direct comparison. The CXR-Age model was developed in screening populations and may behave differently in patients with established cardiac pathology.^18^ Finally, the observational design precludes causal inference.

## Conclusions

Deep learning-derived biological age from pre-operative chest radiographs outperforms EuroSCORE II for predicting short-term mortality across surgical and transcatheter procedures for structural heart disease and is the only score evaluated that discriminates well in both populations. In surgical patients, it matches the more data-intensive STS-PROM score, and in transcatheter patients, it substantially exceeds both clinical scores. As chest radiography is routinely obtained pre-operatively, biological age estimation represents a readily available, automated complement to existing risk scores. Prospective, multicentre validation and integration into multimodal risk-prediction frameworks are warranted.

## Supplementary Material

ztag112_Supplementary_Data

## Data Availability

The data underlying this article cannot be shared publicly due to patient privacy regulations. Requests for data access can be directed to the corresponding author.

## Lead author biography

Era Stambollxhiu is a resident radiologist at TUM Klinikum Rechts der Isar in Munich. She studied at Ludwig Maximilian University of Munich, where she built a strong foundation in medical imaging. Her research centres on the application of artificial intelligence and deep learning in radiology, with a focus on developing models that extract clinically meaningful insights. Additionally, she investigates how large language models can enhance diagnostic workflows and support clinical decision-making.
