# Correction to: Deep learning-derived biological age from preoperative chest radiographs predicts mortality following surgical and transcatheter procedures for structural heart disease beyond EuroSCORE II

**DOI:** 10.1093/ehjdh/ztag177

**Published:** 2026-11-13

This is a correction to: Era Stambollxhiu, Miriam Kumpf, Maximilian-Niklas Bonk, Lisa C Adams, Marcus Makowski, Martin Hadamitzky, Markus Krane, Keno K Bressem, Oliver Deutsch, Deep learning-derived biological age from preoperative chest radiographs predicts mortality following surgical and transcatheter procedures for structural heart disease beyond EuroSCORE II, *European Heart Journal - Digital Health*, Volume 7, Issue 7, August 2026, ztag112, https://doi.org/10.1093/ehjdh/ztag112

In the originally published version of this manuscript, the ‘Funding’ section erroneously indicated that “No external funding was received for the conduct of this study”.

The ‘Funding’ section has been corrected to read:

“This research is funded by the European Union under the Research and Innovation Action (RIA) TWIN-X (Grant Agreement No. 101287679). Views and opinions expressed are, however, those of the authors only and do not necessarily reflect those of the European Union or the granting authority. Neither the European Union nor the granting authority can be held responsible for them”.

