# Supplementary material for: Deep learning-derived biological age from preoperative chest radiographs predicts mortality following surgical and transcatheter procedures for structural heart disease beyond EuroSCORE II
Source: Eur Heart J Digit Health. 2026 Jul 20;7(7):ztag112. doi: 10.1093/ehjdh/ztag112 (PMC13436687; doi:10.1093/ehjdh/ztag112)
Supplement: Supplementary file 1 [file ztag112_supplementary_data.docx]

# Supplementary Tables

**Supplementary Table 1.** Characteristics by Procedure Type

| **Procedure** | **n** | **Age** | **Bio age** | **Delta** | **ES II** | **STS** | **30-d mort** |
| --- | --- | --- | --- | --- | --- | --- | --- |
| TAVI | 515 | 80.4 ± 7.4 | 74.3 ± 5.3 | -6.1 ± 6.1 | 6.8 | 2.9 | 1.4% |
| Mitral ± tricuspid | 248 | 66.1 ± 11.4 | 68.0 ± 7.4 | 1.9 ± 7.7 | 3.4 | 1.1 | 1.2% |
| Aortic valve replacement | 242 | 62.4 ± 11.6 | 66.4 ± 7.8 | 4.0 ± 8.0 | 2.6 | 1.0 | 0.8% |
| Transcatheter mitral | 92 | 77.4 ± 7.6 | 73.0 ± 5.7 | -4.4 ± 6.7 | 6.6 | 3.2 | 3.3% |
| Combined | 59 | 65.7 ± 12.4 | 68.2 ± 7.1 | 2.5 ± 8.3 | 5.9 | - | 5.1% |
| Congenital with CPB | 38 | 53.8 ± 15.0 | 63.7 ± 8.5 | 9.9 ± 9.4 | 2.4 | - | 2.6% |
| Other CPB | 35 | 58.2 ± 14.2 | 65.5 ± 8.1 | 7.3 ± 8.8 | 2.0 | - | 2.9% |
| Transcatheter tricuspid | 29 | 78.6 ± 6.4 | 73.4 ± 4.9 | -5.2 ± 6.0 | 9.0 | - | 3.4% |
| Isolated tricuspid | 8 | 62.9 ± 13.1 | 67.5 ± 7.8 | 4.6 ± 8.2 | 4.1 | - | 0.0% |
| TAVI + transcatheter mitral | 3 | 80.3 ± 4.5 | 74.0 ± 3.6 | -6.3 ± 4.0 | 8.2 | - | 0.0% |

*Values are n, mean ± SD, or %. Bio age = biological age; CPB = cardiopulmonary bypass; ES II = EuroSCORE II (%, mean); STS = STS-PROM (%, mean); TAVI = transcatheter aortic valve implantation. STS-PROM is not defined for several non-TAVI procedure categories and is shown as
“-” where unavailable. Counts sum to 1,269 procedures.*

**Supplementary Table 2.** Age Delta and Resource Utilisation by Subgroup

| **Outcome / subgroup** | **β per year (95% CI)** | **p-value** |
| --- | --- | --- |
| Hospital stay, non-TAVI | 0.09 (0.01 to 0.18) | 0.032 |
| Hospital stay, TAVI | 0.01 (-0.02 to 0.05) | 0.549 |
| ICU stay, non-TAVI | -0.02 (-0.09 to 0.05) | 0.520 |
| ICU stay, TAVI | -0.02 (-0.08 to 0.04) | 0.489 |

*Linear regression of length of stay (days) on age delta (years) within each subgroup. CI = confidence interval; ICU = intensive care unit; TAVI = transcatheter aortic valve implantation.*

**Supplementary Table 3.** Calibration Metrics for 30-Day Mortality Models by Subgroup

| **Subgroup / model** | **Brier score** | **Scaled Brier** |
| --- | --- | --- |
| Non-TAVI |  |  |
| EuroSCORE II | 0.0108 | -0.002 |
| STS-PROM | 0.0045 | 0.583 |
| CXR-Age | 0.0097 | 0.100 |
| TAVI |  |  |
| EuroSCORE II | 0.0138 | -0.001 |
| STS-PROM | 0.0138 | 0.000 |
| CXR-Age | 0.0136 | 0.010 |

*Lower Brier score indicates better prediction; scaled Brier 1 = perfect, 0 = uninformative. Calibration intercepts were approximately 0 and slopes approximately 1 across models, indicating adequate calibration. CXR-Age = deep learning biological age from chest radiographs; TAVI = transcatheter aortic valve implantation.*

**Supplementary Table 4.** Internal (Bootstrap) Validation of Discrimination by Subgroup

| **Model** | **Non-TAVI apparent / corrected** | **TAVI apparent / corrected** |
| --- | --- | --- |
| EuroSCORE II | 0.770 / 0.769 | 0.736 / 0.749 |
| STS-PROM | 0.945 / 0.932 | 0.717 / 0.724 |
| Biological age | 0.868 / 0.866 | 0.958 / 0.957 |
| Chronological age | 0.780 / 0.800 | 0.871 / 0.869 |
| EuroSCORE II + biological age | 0.875 / 0.863 | 0.959 / 0.949 |
| STS-PROM + biological age | 0.922 / 0.910 | 0.958 / 0.955 |

*Apparent and optimism-corrected AUC from 1,000 bootstrap resamples. TAVI = transcatheter aortic valve implantation.*

**Supplementary Table 5.** Likelihood-Ratio Tests for Incremental Value (30-Day Mortality)

| **Comparison** | **Non-TAVI χ², p** | **TAVI χ², p** |
| --- | --- | --- |
| CXR-Age added to EuroSCORE II | 13.48, <0.001 | 21.06, <0.001 |
| CXR-Age added to STS-PROM | 0.88, 0.348 | 23.32, <0.001 |
| EuroSCORE II added to CXR-Age | 0.24, 0.627 | 0.16, 0.690 |
| STS-PROM added to CXR-Age | 22.00, <0.001 | 0.50, 0.481 |

*Likelihood-ratio test chi-square statistics (1 df) and p-values for adding one predictor to a model already containing the other. CXR-Age = deep learning biological age from chest radiographs; TAVI = transcatheter aortic valve implantation.*

# Figure Legends

**Figure 1.** Bland-Altman plot showing agreement between biological and chronological age across the full cohort. Horizontal lines indicate the mean difference (-1.1 years) and the 95% limits of agreement (-17.5 to +15.2 years).

**Figure 2.** Receiver operating characteristic curves for 30-day mortality by procedural subgroup. (A) Non-TAVI subgroup and (B) TAVI subgroup, comparing EuroSCORE II, STS-PROM and biological age (CXR-Age). Biological age was the strongest single predictor in the TAVI subgroup, whereas STS-PROM was strongest in the non-TAVI subgroup.

**Figure 3.** Hospital and ICU length of stay by age-delta category, shown separately for the non-TAVI and TAVI subgroups. The association between higher age delta and longer hospital stay was confined to the non-TAVI subgroup.

**Supplementary Figure 1.** Distribution of chronological age, biological age, and age delta in the study cohort.

**Supplementary Figure 2.** Correlation between biological and chronological age (r = 0.77). The dashed line represents the identity line (y = x); the solid line represents the linear regression fit.

**Supplementary Figure 3.** Receiver operating characteristic curves for 90-day mortality by procedural subgroup. (A) Non-TAVI subgroup and (B) TAVI subgroup.

**Supplementary Figure 4.** Receiver operating characteristic curves for combined (bivariable) models by subgroup and timepoint: (A) non-TAVI 30-day, (B) TAVI 30-day, (C) non-TAVI 90-day, and (D) TAVI 90-day mortality.

**Supplementary Figure 5.** Thirty-day and 90-day mortality rates stratified by age-delta category.

**Supplementary Figure 6.** Postoperative major-complication rates stratified by age-delta category.

# Supplementary Figures

**Supplementary Figure 1.** Distribution of chronological age, biological age, and age delta.

**Supplementary Figure 2.** Correlation between biological and chronological age.

**Supplementary Figure 3.** Receiver operating characteristic curves for 90-day mortality by subgroup.

**(A) Non-TAVI**

**(B) TAVI**

**Supplementary Figure 4.** Receiver operating characteristic curves for combined (bivariable) models by subgroup and timepoint.

**(A) Non-TAVI, 30-day**

**(B) TAVI, 30-day**

**(C) Non-TAVI, 90-day**

**(D) TAVI, 90-day**

**Supplementary Figure 5.** Thirty-day and 90-day mortality by age-delta category.

**Supplementary Figure 6.** Postoperative major-complication rates by age-delta category.
